# Clinical Applications of Mesenchymal Stem Cells in Chronic Diseases

**DOI:** 10.1155/2014/306573

**Published:** 2014-04-30

**Authors:** Andrea Farini, Clementina Sitzia, Silvia Erratico, Mirella Meregalli, Yvan Torrente

**Affiliations:** Laboratorio di Cellule Staminali, Dipartimento di Fisiopatologia Medico-Chirurgica e dei Trapianti, Università degli Studi di Milano, Fondazione IRCCS Ca' Granda Ospedale Maggiore Policlinico, Centro Dino Ferrari, Via F. Sforza 35, 20122 Milano, Italy

## Abstract

Extraordinary progress in understanding several key features of stem cells has been made in the last ten years, including definition of the niche, and identification of signals regulating mobilization and homing as well as partial understanding of the mechanisms controlling self-renewal, commitment, and differentiation. This progress produced invaluable tools for the development of rational cell therapy protocols that have yielded positive results in preclinical models of genetic and acquired diseases and, in several cases, have entered clinical experimentation with positive outcome. Adult mesenchymal stem cells (MSCs) are nonhematopoietic cells with multilineage potential to differentiate into various tissues of mesodermal origin. They can be isolated from bone marrow and other tissues and have the capacity to extensively proliferate *in vitro*. Moreover, MSCs have also been shown to produce anti-inflammatory molecules which can modulate humoral and cellular immune responses. Considering their regenerative potential and immunoregulatory effect, MSC therapy is a promising tool in the treatment of degenerative, inflammatory, and autoimmune diseases. It is obvious that much work remains to be done to increase our knowledge of the mechanisms regulating development, homeostasis, and tissue repair and thus to provide new tools to implement the efficacy of cell therapy trials.

## 1. Introduction

Since the work of Friedenstein, that firstly described bone marrow- (BM-) derived stromal cells with the capacity of differentiation into bone [1], it was thought that nonhaematopoietic stem cell resided in the bone marrow, the so-called mesenchymal stem cells (MSCs) [2, 3]. The group of Caplan identified the MSCs from BM for the expression of the specific antigen markers CD105 and CD73 [4]. Pittenger defined the MSCs as multipotent stem cell with the ability to differentiate into adipose tissue, bone, and cartilage [5]. According to this multilineage differentiation potential, it was believed that MSCs mediated tissue and organ repair [6, 7]. However, further studies assessed that, following specific molecular cues, MSCs reached the site of injury and allowed the repair of tissues by means of the expression of different trophic factors [8–10]. In the last 20 years, MSCs were isolated from a wide range of tissues [11–14] and organs [15, 16]. Furthermore, it was demonstrated that under specific stimuli MSCs possessed an incredible capacity of transdifferentiation, developing in mesodermal (myocyte, osteocyte, endothelium, adipocyte, and cardiomyocyte), ectodermal (neuronal), and endodermal (hepatic, pancreatic, respiratory epithelium) lineages. In the presence of *β*-glycerol-phosphate, ascorbic acid-2-phosphate, dexamethasone, and fetal bovine serum, MSCs proliferated as osteoblasts. On the other side, when they were grown with a serum-free nutrient medium added with TFG- *β* or family-related molecules, MSCs proliferated as chondrocytes, expressing cartilage-specific extracellular matrix components [17]. Similarly, it could be possible to induce the formation of adipocytes by means of peroxisome proliferator-activated receptor-*γ* (PPAR-*γ*), fatty acid synthetase, and isobutylmethylxanthine while, on the contrary, IL-1 and TNF-*α* blocked MSCs-adipogenetic differentiation. As published by Barry and Murphy, MSCs differentiation into myoblasts was driven by 5-azacytidine and amphotericin B [17]. Recently, different works suggested that MSCs were strictly associated with vessels and possibly with pericytes, the perivascular cells that surround microvessels [18]. It was demonstrated that pericytes retained the ability to differentiate not only into osteoblasts, adipocytes, and fibroblasts but also into neural lineages if cultivated with bFGF [19] and into smooth muscle cells if stimulated with low concentration of oxygen [20]. It is well known that MSCs are able to express integrins, adhesion molecules, and chemokine receptors that regulate their capacity of migration and homing: CCR1, CCR4, CCR7, CCR10, and CXCR5 [4, 21]. Thanks to the expression of these molecules, MSCs can reach damaged tissues through endothelial cell layers and participate not only in tissue regeneration but also in BM microenvironment replenishment [22]. Stromal derived factor (SDF)-1 is associated with mobilization of stem cells into the periphery and homing to the site of injury [23, 24]: it was showed that in diverse tissue injures SDF-1 functions as a MSCs chemoattractant [25–28]. According to these evidences, MSCs were evaluated in several studies for their safety and efficacy of transplantation. Studies published by Gao and Herrera confirmed the ability of MSCs to engraft into various organs following transplantation (liver, bone, and lung) [29, 30], while the groups of Jackson and Orlic successfully used them in the preparation of infarcted myocardium [31–33]. Furthermore, MSCs were noted to enhance angiogenesis in the myocardium [34] and also to allow the reduction of myocardial fibrotic area, probably due to their capacity of increasing the capillary density [35]. Hofstetter and colleagues demonstrated that MSCs exert their role also indirectly, enhancing the expression of growth factors that allowed the regeneration of damaged tissues [36]. However, further studies are necessary to better identify (i) all the molecules other than chemokines and adhesion molecules that drive MSCs to the site of injury; (ii) growth media to obtain reproducible culture techniques and to enhance safety of expanded MSCs; (iii) host responses to allogenic MSC therapy.

## 2. MSCs Isolation 

Citofluorimetric analysis performed on MSCs showed that they express CD44, CD73, CD90, and CD105 receptors while lacking hematopoietic stem cell markers such as CD14, CD31, CD33, CD34, and CD45. Due to the absence of specific mesenchymal cell markers and the heterogeneity of the MSC populations, the Mesenchymal and Tissue Stem Cell Committee of the International Society for Cellular Therapy (ISCT) established three minimal criteria that MSCs isolated from human bone marrow and other mesenchymal tissues must have* in vitro*: (i) adherence to plastic in standard culture conditions, (ii) display of a specific surface antigen expression pattern (CD73+ CD90+ CD105+ CD34− CD45− CD11b− CD14− CD19− CD79a− HLA-DR−), and (iii) multipotency, that is, differentiation potential along the osteogenic, chondrogenic, and adipogenic lineages [37]. The heterogeneity of the MSC population is demonstrated by* in vitro* differentiation assays, where most of the population showed a differentiation potential towards the classical three cell types.

## 3. Immunomodulatory Effects of MSCs 

Several studies have demonstrated that MSCs can inhibit cytotoxic T cells and natural killer (NK) cells [38, 39] by means of different pathways. MSCs can exert their immunomodulatory functions by secreting suppressors of T-cell development (TGF*β* and hepatocyte growth factor (HGF)) [40] and proliferation such as leukemia inhibitory factor (LIF) [41] and IFN-*γ* [42]. Furthermore, MSCs can induce the expression of TNF-*α* and IL-1 leading to unbalanced secretion of chemokines and inducible nitric oxide (iNOS) [43]. More interestingly, the works of Spaggiari et al. [44] and Poggi et al. [45] showed that MSCs isolated from BM are not recognized by NKs as they express human leukocyte antigen (HLA) class I molecules. This way, MSCs were seen as the most feasible population of stem cells for cell transplantation experiments. Otherwise, recent studies demonstrated that MSCs were efficiently lysed by the cytotoxic immune effectors [39, 46]. The work of Jewett et al. showed that IL-2 treated NKs recognized and destroyed MSCs while IFN-*γ* had the opposite effect [47]. As the IFN-*γ* is secreted by monocytes, the authors postulated that these cells not only served as protector of MSCs but also allowed the differentiation of stem cells by NF*κ*B dependent and independent pathways [47]. Giuliani et al. showed that MSCs expressed functional Toll-like receptors (TLR) that promote their proliferation and cytokine secretion [48]. They also identified a molecule, called MICA, that formed a complex with other immunoregulatory proteins and together with TLRs ligands protected MSCs against NKs aggression [48].

## 4. MSCs and Chronic Diseases

### 4.1. MSCs and Musculoskeletal Diseases

As for the other tissues previously described, MSCs were isolated from human adult skeletal muscle [49, 50]. In addition, Gonçalves described the ability of MSCs to complement dystrophin deficiency [51], and Németh et al. showed that MSCs increased the survival rate of model animals by the modulation of macrophage activity [52]. For these reasons, these cells became feasible to therapeutic application for Duchenne muscular dystrophy (DMD). Different groups demonstrated that transplantation of MSCs into murine model of DMD replenished the host satellite cell compartment—allowing the expression of dystrophin and ameliorating the dystrophic phenotype—and also remained as a pool of quiescent satellite cells [53, 54]. Similarly, de Bari and coworkers isolated MSCs from human synovial membrane and injected them into mdx mice, showing MSCs persistence into host muscles up to six months [55]. Gang et al. demonstrated that MSCs derived from human umbilical cord blood (UCB-MSCs) differentiated into skeletal muscle and expressed MyoD and myogenin, muscle-specific transcription factors: transplanted into dystrophic mice, they allowed highly detectable expression of myosin [56].

The role that MSCs play in regulating inflammation is now clear. It is a “multistep” event as MSCs could exert their role as a negative controller/suppressor, by expressing SDF-1 and CCL2 [57], by inhibiting macrophage activation [58] or by Th1, NK, and cytotoxic T-cell generation [39]. Alternatively, MSCs could act as positive controller/activator by enhancing the proliferation of Th2 cells and regulatory T cells (Treg) [59] and by the expression of immune suppressive cytokines and enzymes [60, 61]. Given these evidences, together with the fact that the only functional treatment for DMD is the corticosteroid therapy that regulates the inflammatory reactions, MSCs were widely used in dystrophic animal models. Firstly, MSCs were injected into the uterus of mdx mice at different days of pregnancy: the cells were observed to engraft in different muscles but their functionality was not altered [62, 63]. Adipose-derived MSCs (AD-MSCs) were transplanted into dystrophic mice and they homed to necrotic fibers. Moreover, AD-MSCs allowed the re-expression of dystrophin and muscular remodelling, even if at lower rate [64]. Furthermore, injection of MSCs was seen to inhibit the expression of creatine kinases whereas increasing the number of centrally nucleated myofibers [65]. According to these evidences, Kong et al. transplanted UCB-MSCs into animal model of limb girdle muscular dystrophies (LGMDs), characterized by predominant weakness and wasting of proximal muscles, but they did not obtain promising results [66]. Although the bones naturally restore without significant scarring, infections, trauma, and cancer could impair their functional restoration, causing several bone defects [67, 68]. Cell-based therapies need to isolate MSCs from the BM of the patient, to expand and enrich the cells and to seed them into the most suitable three-dimensional scaffold and/or matrix [69]. As an example, osteonecrosis is caused by femoral death due to poor blood supply [70]: three patients were treated with MSCs infusion with TCP-treated matrix and good results were obtained [71]. Similarly, Nöth et al. injected a preparation of MSCs into three patients and obtained encouraging results, as shown by radiographic and magnetic resonance imaging examination [72]. MSCs were also successfully used for spinal fusion disease [73], so that phase I clinical trials arose [74, 75]. Patients affected by severe osteogenesis imperfecta were injected systematically with purified allogenic MSCs: these cells were able to engraft into host bones, where they proliferated into osteoblasts and allowed an amelioration in the total bone mineral density [76, 77]. Although these encouraging studies were performed, the amount of MSCs recruited into the bones was too small in a clinical point of view. Alternatively, the group of Le Blanc treated a female fetus with multiple intrauterine fractures with allogenic fetal MSC [13].

### 4.2. MSCs and Cardiovascular Diseases

Starting from the evidences that MSCs not only secreted molecules that exerted important effects on cellular microenvironment [36] but also differentiated* in vitro* into cardiomyocytes [78, 79], these cells were extensively used for cardiovascular repair. Shake and Nagaya demonstrated that, following systemically injection into rodent models of these diseases, MSCs engrafted and partially repaired the infarcted myocardium [80, 81]. In particular, Nagaya and collaborators showed that transplanted MSCs increased capillary density and decreased the collagen volume fraction and the fibrosis in the myocardium of a rat suffering from dilated cardiomiopathy [82]. Furthermore, they also noted a significant ventricular functional recovery as previously demonstrated [83]. According to these promising evidences, Katritsis et al. treated 11 infarcted patients with autologous MSCs, together with endothelial progenitor's cells, and showed partial improvement of myocardial contractility. Unfortunately, they were not able to decipher the mechanisms responsible for these phenomena [84]. Similarly, several infarcted patients that were subjected to coronary intervention were transplanted with autologous MSCs that improved left ventricular function [85]. Takahashi and collaborators assessed that the molecules secreted by MSCs were able to protect the myocardium by preserving its contractile capacity; in particular, MSCs-derived cytokines inhibited the apoptosis of cardiomyocytes, allowing the formation of new vessels in damaged tissues [86].

### 4.3. MSCs and Liver Disease

Fulminant hepatic failure (FHF) is a severe disease characterized by massive hepatocellular death: the only treatment is liver transplantation that requires lifelong immunosuppression and high costs. Different works demonstrated that MSCs-secreted molecules not only allowed tissue repair of infarcted tissue [82] but furthermore prevented parenchymal cell loss [87]. This way, van Poll and colleagues reported that, following systemic injection of MSCs in a rat model for FHF, there was an amelioration of the pathological phenotype—so that liver injury biomarkers were not released—and, more interestingly, hepatocellular death was drastically reduced, while hepatocytes proliferation increased [88]. Concerning cirrhosis, four patients were injected with autologous MSCs in a phase I trial; they did not suffer from any side effects, thus improving their quality of life [89]. Similarly, 8 patients with end-stage liver disease were treated with MSCs and their condition ameliorated demonstrating that this treatment could be feasible and efficient for this kind of pathologies [90].

### 4.4. MSCs and Autoimmune Diseases

Since Riordan et al. suggested that in the bone marrow one of the most important functions of MSCs could be the protection of haematopoietic precursor from inflammatory damage [91], the use of MSCs as inhibitors of inflammation became conceptually appealing. This way, MSCs were used to block the development of chronic inflammatory processes that are typical of DMD (as described in detail in Section 4.1), autoimmune arthritis, diabetes, and lupus.

#### 4.4.1. Rheumatoid Arthritis

Rheumatoid arthritis (RA) is characterized by chronic joint inflammation due to loss of immunologic self-tolerance. González and colleagues injected DBA/1 mice that suffered from collagen-induced arthritis, with MSCs derived from human adipose tissue, and evaluated the inflammatory response of treated animals [92]. They showed that following the injection of AD-MSCs the levels of inflammatory cytokines and chemokines were decreased as the expansion of antigen-specific Th1/Th17 cell. In contrast this treatment increased the production of IL-10. Together with its well-known function as an anti-inflammatory factor [93], recent findings demonstrated that IL-10 is fundamental for the development of Tregs that control self-antigen–reactive T cells and induce peripheral tolerance* in vivo* [94]. Interestingly, they found that treated mice had an increase in the percentage of CD4+ CD25+ FoxP3+ Tregs and suggested that these cells could migrate to the joints, regulating the suppression of the self-reactive response [92]. It is known that type-II collagen (CII), one of the components of hyaline cartilage, acts as an autoantigen in RA. When CII and the other antigenic peptides are recognized by T cells, they cause the uncontrolled activation of immune system cells, leading to destruction of the joints typical of RA patients. Zheng et al. demonstrated that MSCs isolated from RA patients exerted immunosuppressive functions, by inhibiting T-cell proliferation, blocking the secretion of several proinflammatory cytokines, and allowing the expression of anti-inflammatory IL-10 [95]. They also obtained MSCs from chondrocytes and described that, following transplantation into RA joints, these cells not only suppressed the inflammation regulating the secretion of TGF- *β* 1 but also prevented joints destruction [95].

Similar to the previously described work of González et al. [92], experimental data from Augello [96] and Mao [97] confirmed the positive effects of MSCs transplantation into animal model of collagen-induced arthritis while others did not describe any amelioration [98, 99]. Schurgers et al. showed the discrepancy between the* in vitro* and* in vivo* immunosuppression ability of MSCs.* In vitro*, MSCs inhibited the proliferation of T cells by regulating the levels of IFN-*γ* whereas* in vivo* transplantation of these cells into CIA animal models did not affect the progression of the disease [100]. As an explanation, due to problems during intravenously injection, MSCs could not reach the spleen and lymph nodes, so that they did not exert their functions. As MSC treatment for this pathology was not efficacious, they also suggested to focus on Treg as in mice injected with these cells the pathological signs of arthritis were significantly ameliorated [101, 102]. Another work from Bouffi et al. demonstrated that MSCs elicited their immunosuppressive effect by means of a pathway regulated by the prostaglandine-2. Moreover, they showed that MSCs operated independently from Treg cell induction. Finally they suggested that the contradictory effect of MSCs transplantation could be related to the different age of the mice used in those studies [103].

#### 4.4.2. Systemic Lupus Erythematosus

Systemic lupus erythematosus (SLE) is an autoimmune disease that can affect any part of the body [104]. Recent findings demonstrated several defects in the hematopoietic system of SLE patients, probably due to unbalanced expression of cytokines and other growth factors. Interestingly, it was found that bone marrow-derived CD34+ stem cells overexpressed different surface markers such as CD123 and CD166 that are closely related to T-cell development inflammation [105]. Accordingly, Kushida et al. showed that transplantation of hematopoietic stem cells prevented the onset of the disease in the most commonly studied mouse model of SLE, the MRL/lpr mice [106]. Sun and colleagues determined a possible role of BM-derived MSCs in the haematological disorder typical of SLE patients [107] and suggested that MSCs transplantation could be used to ameliorate the autoimmune progression of the disease [108]. In fact they found that MSCs inhibited T-lymphocytes and Th2 proliferation and B-cell production of autoantibody, so that the pathological signs of MRL/lpr mice were drastically diminished [108].

#### 4.4.3. Type 1 Diabetes

Type 1 diabetes is an autoimmune disease mediated by the production of auto-antibody directed against the *β*-cells of the pancreas. As a consequence of the destruction of these cells, the quantity of insulin produced is not sufficient to control sugar blood level. Despite the exogenous administration of insulin, long-term consequences of hyperglycemia usually occur, including vascular degeneration, blindness, and kidney failure. Islet replacement is the best way to fully reproduce the physiological release of insulin; however, both the limited availability of transplantable organs and the need for immunosuppression have limited the application of this strategy [109]. Recently it has been suggested that MSCs can overcome these problems as they can be differentiated into glucose-responsive, insulin-producing cells and they possess immunomodulatory properties. It was hypothesized that resident pancreatic MSCs could be forced to adopt a pancreatic fate* in vitro*. Thus, Zulewski et al. reported the isolation of nestin-positive islet-derived progenitor cells from rat pancreatic islets and their ability to differentiate* in vitro* toward pancreatic endocrine phenotype [110]. Similarly Huang and Tang described the correction of hyperglycemia in diabetic NOD-SCID mice thanks to nestin-positive precursors derived from human fetal pancreas [111]. However, the results of these studies were controversial and partially inconclusive [112–115] so that MSCs from other tissues could be an alternative. Among them, bone marrow derived MSCs were shown to partially differentiate into endocrine pancreatic cells [116]; furthermore,* in vivo* maturation of these cells partially compensated their low differentiation efficiency* in vitro* [117]. An intriguing option comes from studies on umbilical cord blood-derived MSCs that demonstrated the expression of pancreatic development genes in these cells. Recently a population of UCB-derived cells was shown to behave like hES cells, recapitulating the same differentiation steps from early stages to *β*-cells [118]. In conclusion, before MSCs clinical application in diabetes further studies are needed to improve MSCs based protocols and above all to expand our knowledge on MSCs immunogenicity in a HLA-mismatched context.

### 4.5. MSCs and Neurodegenerative Diseases

#### 4.5.1. Multiple Sclerosis

Multiple sclerosis is an important cause of neurological disability in young adults. Although it is a multifactorial disease, it is known that the presence of an aberrant immunoresponse leads to patches of damage throughout the brain and spinal cord. Autoreactive T cells cause myelin destruction and secondary oligodendrocyte and axonal damage [119]. Despite the efficacy of immunomodulatory or immunosuppressive drugs in controlling the number of relapses, no current therapy is effective to arrest the progressive phase of the disease. The therapeutic potential of stem cell lies in enhancing myelin regeneration, through the replacement of lost oligodendrocytes, and therefore in preserving axons, thanks to the neurotrophic support [120]. However, the widespread distribution of lesions and the gray matter damage render the therapeutic efficacy of direct mesenchymal injection really low. Furthermore bone marrow-derived cells ability to make oligodendrocytes was low [121]. Nevertheless, bone marrow-derived MSCs have pronounced immune-modulating and immunosuppressive properties [122, 123] so as they were being tested in clinical trial for relapsing-remitting multiple sclerosis Mesenchymal Stem Cells for Multiple Sclerosis (MESEMS) (NCT01854957). Furthermore it was thought that mesenchymal stem cells promote self-repair by reducing scar formation, by stimulating the formation of new blood vessel, and by secreting growth and neuroprotective factors, such as superoxide dismutase-3 [124]. After intravenous injection, many cells entered the CNS and became widely distributed both in experimental models and in patients [125–127]: safety studies did not evidence adverse effect such as tumor formation, except for meningeal syndrome and some preliminary evidence of beneficial effects that were reported [128, 129].

#### 4.5.2. Amyotrophic Lateral Sclerosis

Suzuki et al. isolated MSCs from muscles and genetically modified them to constitutively express glial-derived neurotrophic factor (GDNF). Then, they transplanted the engineered MSCs into rat model of amyotrophic lateral sclerosis (ALS), a neurodegenerative disease in which patients lose motor neurons and suffer from progressive and lethal paralysis. Interestingly, ALS rats ameliorated the pathological phenotype, increasing the number of neuromuscular connections [130].

#### 4.5.3. Parkinson's Disease

Parkinson's disease (PD) is a neurodegenerative disorder characterized by the progressive loss of dopaminergic neurons (DA), especially in the pars compacta of the substantia nigra. The mesostriatal dopaminergic pathway projects in the striatum and their absence causes several motor complications, including rigidity, bradykinesia, and postural instability [131, 132]. DA agonists and Levodopa (l-dopa) are effective symptomatic therapy, but unfortunately, with long-term use, they become inefficient and patients develop significant side effects. Stem cell therapy, with the aim of replacing lost neurons, is the most promising strategy for this disease [133]. It has been demonstrated that MSCs cells can enhance the levels of tyrosine hydroxylase (TH) and dopamine levels after transplantation in PD animal models [134]. Furthermore it has been suggested that these cells contribute to neuroprotection by secreting trophic factors, like EGF, VEGF, NT3, FGF-2, HGF, and BDNF or through antiapoptotic signalling [135] without differentiation in neuronal phenotype [136]. For these reasons new strategies, involving the genetic modification of hMSCs, arose, as a tool to induce the secretion of specific factors or to increase the percentage of DA cell differentiation [137]. For example Barzilay et al. transduced adult-derived bone marrow MSCs with a lentivirus carrying LMX1a gene: these cells showed an expression profile similar to a developing mesodiencephalic neuron and allowed DA cell differentiation [138].

#### 4.5.4. Alzheimer Disease

Alzheimer disease (AD) is the most common form of neurodegenerative dementia; affected patients suffer from progressive loss of memory and intellectual abilities. Major anatomopathological features of AD are represented by *β*-amyloid deposition and neurofibrillary tangles formation that ultimately end in cholinergic neurons degeneration. No treatment is currently able to stop the progression of AD [139]. Recently, different studies tried to ameliorate neuropathological deficits in animal model of Alzheimer's disease through stem cell therapy. In particular, Shin et al. focused on clearance of amyloid plaque and they demonstrated that MSCs could enhance the cell autophagy pathway increasing neuron survival both* in vitro* and* in vivo* [140]. Similarly Ma's group demonstrated that adipose-derived MSC, once transplanted in AD model mice, could modulate the inflammatory environment; in particular they caused an activation of the microglia that promoted the expression of alternative markers and A*β*-degrading enzymes, while decreasing expression levels of proinflammatory factors [141]. Following promising results from MSCs treatment for autoimmune diseases, it was thought to modulate the inflammatory environment of AD. In particular abnormalities of Tregs in cell number and/or function were observed [142] and it was shown that they could modulate microglial activation [143]. Yang et al. demonstrated that umbilical cord-derived MSCs activated Tregs* in vitro* and once transplanted in AD animal model, Tregs modulated microglia activation, increasing neuron survival [144].

## 5. Clinical Applications of MSCs

Before clinical applications of stem cells we need to understand their biological characteristics in order to obtain therapeutic effects. In case of MSCs, four properties are considered the most important to guarantee a clinical rescue: (i) the ability to home to the site of inflammation, following tissue injury, when injected intravenously; (ii) the ability to differentiate into various cell types; (iii) the ability to secrete multiple bioactive molecules capable of stimulating recovery of injured cells and of inhibiting inflammation; (iv) the lack of immunogenicity and the ability to perform immune-modulatory functions [87]. Moreover, the role of MSCs in therapeutic effects has still to be elucidated. MSCs have the capacity to migrate and to engraft in site of inflammation, after local or systemic administration. Various studies demonstrated that, under a variety of pathologic conditions, MSCs selectively home to sites of injury, indifferently from the tissue. Ortiz et al. showed that murine MSCs could home to lung in response to injury, adopt an epithelium-like phenotype, and reduce inflammation in lung tissue of mice challenged with bleomycin [145]. Liu et al. found that transplanted MSCs could migrate to injured muscle tissues in mdx mice [64]. Cell migration depends on many signals, including growth factors, interleukins, and chemokines, secreted by injured cells and immune cells [146]. Recently Yagi et al. demonstrated that the migration of MSCs is under the control of many tyrosine kinase growth factor receptors like platelet-derived growth factor (PDGF) and insulin like growth factor 1 (IGF-1); in addition, several chemokines such as CCR2, CCR3, CCR4, or CCL5 ameliorate MSCs migration in* in vitro* migration assays [147].

The first clinical trial using culture-expanded MSCs was performed in 1995 and 15 patients were treated with autologous stem cells [148]. After the first one, a number of clinical trials have been conducted to test the feasibility and efficacy of MSCs therapy. From 2011, 206 clinical trials using MSCs were published on the public clinical trials database (http://www.clinicaltrials.gov/) showing a very wide range of therapeutic applications. Most of these trials are Phase I studies, Phase II, and a combination of Phase I/II studies. Only a small number of these trials are in Phase III or Phase II /III. Most of the trials reported lack of adverse effects in the medium timing, although few of them showed mild and transient peri-injection effects: in general, MSCs seem to be well tolerated [87]. Very promising results were obtained by the injection of autologous and allogenic MSCs in patients suffering from osteogenesis imperfecta [76] while* in vitro* expanded MSCs were used to treat severe [149] and treatment-resistant [150, 151] GVHD patients. In addition, many completed clinical trials have demonstrated the efficacy of MSC infusion for diseases including acute myocardial ischemia (AMI), stroke, amyotrophic lateral sclerosis (ALS), and muscular dystrophies.

## 6. Conclusions

MSCs have many characteristics required for an optimal cell source for cell-replacement therapies, as they are easy to isolate, and retain the ability to expand over a long period of time without serious technical problem. MSCs are linearly restricted; however, there is evidence that MSCs* in vitro* can also express property of ectodermal cells [12]. One requisite of the stem cell-based therapeutic approach is to replace damaged cells. For example, in PD, many studies have focused on examining whether cells replacement therapy could be used. Although transplanted MSCs showed a low cell replacement potential, they improve the environment through the release of neuroprotective factors and they can be engineered to ensure specific expression and secretion. Moreover, MSCs promote “bystander” immunomodulation, as they can release soluble molecules and express immunorelevant receptors that are able to modify the inflammatory environment. However, many questions have to be answered both from preclinical and clinical studies using MSCs before MSCs can be used in wider clinical practice. First of all the safety: now, after MSC administration, few adverse effects have been described in terms of immediate and late effects. The relatively small number of treated patients does not permit to draw definitive conclusions on the safety of MSCs. Unfortunately, MSCs have been reported to promote tumor growth [152] and metastases [153]. Prockop et al. described that MSCs cultured with the clinical cell-therapy protocols commonly used showed potential tumorigenic transformation [154]. Chen et al. found that MSCs could aggravate arthritis in collagen-induced arthritis model by at least upregulating secretion of IL-6, which favors Th17 differentiation [155]. Secondly, quality control:* in vitro* cell amplification needs bacteriological tests (mainly in liquid medium) to face contaminations. In addition, viability and phenotype tests, oncogenic tests, and endotoxin assay should also be included. For each disease type and severity, optimal timing of MSCs administration, cell dose, and schedule of administration need to be decided. Third, clinical grade production: clinical application of MSC requires a large number of cells, so* in vitro* expansion of MSC is necessary. Studies have suggested that continuous passaging of MSCs could lead to cell transformation. Bernardo et al. found that MSCs expansion* in vitro* can be safety until passage 25 [156]. Fourth, clinical transition: it is obvious that much work remains to be done to increase our knowledge of the mechanisms regulating development, homeostasis, and tissue repair and thus provide new tools to implement the efficacy of cell therapy trials. Additionally, there is an urgent requirement to address transplantation related issues, such as engraftment, angiogenesis, tissue remodeling, and modulation of the immune response. Currently, more randomized, controlled, multicenter clinical trials are needed to find the optimal conditions for MSC therapy. In general, we think that successful cell therapy necessitates continuous interaction among biologists, clinicians, and patient working groups in the context of different tissues and diseases.
